# Internalizing problems and suffering due to sensory symptoms in children and adolescents with and without autism spectrum disorder

**DOI:** 10.3389/fpsyg.2022.872185

**Published:** 2022-08-05

**Authors:** Yurika Tsuji, Shu Imaizumi, Masumi Sugawara, Arata Oiji

**Affiliations:** ^1^Graduate School of Humanities and Sciences, Ochanomizu University, Tokyo, Japan; ^2^Institute for Education and Human Development, Ochanomizu University, Tokyo, Japan; ^3^Faculty of Human Studies, Shirayuri University, Tokyo, Japan; ^4^Graduate School of Medical Science, Kitasato University, Kanagawa, Japan

**Keywords:** autism spectrum disorder, sensory symptoms, sensory profile, suffering, internalizing problems

## Abstract

Sensory symptoms are common in autism spectrum disorder (ASD). Previous studies have shown a positive correlation between sensory symptoms and internalizing problems; however, the role of the suffering due to sensory symptoms is not well understood. In the present study, we hypothesized that the relationship between sensory symptoms and internalizing problems in children is mediated by children’s and surrounding people’s suffering due to sensory symptoms. Parents of 113 students aged 6–15 years with and without ASD completed questionnaires about their children’s autistic traits, sensory symptoms, suffering due to sensory symptoms, and internalizing problems. The results showed that autistic traits and sensory symptoms were distributed as a continuum throughout children with and without ASD. Therefore, we investigated the relationship among variables in children with and without ASD attending regular classes. Structural equation modeling indicated that those who scored higher on sensory symptoms demonstrated greater suffering due to sensory symptoms as predictors of internalizing problems. Our findings provide evidence for developing a support system that specifically reduces suffering due to sensory symptoms, especially for students in regular classes.

## Introduction

In the fifth edition of the Diagnostic and Statistical Manual of Mental Disorders, the subtypes of pervasive developmental disorders were combined into a single category of autism spectrum disorder (ASD). Individuals with ASD have persistent deficits in social communication and interaction across multiple contexts, and restricted or repetitive patterns of behavior (American Psychiatric Association, 2013). The Autism-Spectrum Quotient (Baron-Cohen et al., 2001) and Social Responsiveness Scale (Constantino and Gruber, 2005) were developed to measure the degree of autistic traits in individuals. These traits are distributed as a continuum in the general population, and there is no natural ‘cutoff’ to differentiate between children with and without ASD (Kamio et al., 2013a). Therefore, individuals with strong autistic traits who are not diagnosed with ASD may still require support, and the risk of secondary problems increases if they cannot access it.

Moreover, individuals with ASD exhibit more significant anxiety than those without ASD. They often develop co-occurring disorders, such as anxiety and mood disorders (De Bruin et al., 2007; White et al., 2009). Anxiety and depression are included in internalizing problems characterized by intropunitive emotions and moods (Zahn-Waxler et al., 2000), and autistic traits have been associated with internalizing problems in children without ASD (Hallett et al., 2010; Kamio et al., 2013b). Thus, higher autistic traits indicate greater internalizing problems even in the general population.

Among autistic traits, sensory symptoms have gained attention in recent years. Many individuals with ASD have sensory symptoms (Bromley et al., 2004; Leekam et al., 2007; Crane et al., 2009) and have significantly more sensory symptoms than typical developing (TD) children (Rogers et al., 2003; Leekam et al., 2007; McCormick et al., 2016). Although children with developmental disorders other than ASD also have sensory symptoms, some were peculiar to children with ASD (Cheung and Siu, 2009; van der Linde et al., 2013; McCormick et al., 2016). On the other hand, people who have not been diagnosed with ASD, but have high levels of autistic traits, may have sensory symptoms (Robertson and Simmons, 2013).

Sensory symptoms are also associated with internalizing problems in individuals with ASD or high levels of autistic traits. For example, some studies demonstrated the relationship between sensory symptoms and internalizing problems in children with ASD (Pfeiffer et al., 2005; Tsuji et al., 2009; Mazurek et al., 2013). Moreover, the relationship between sensory symptoms and anxiety has been reported in adults, most of whom did not have ASD (Horder et al., 2014).

Although the mechanism of the relationship between sensory symptoms and internalizing problems has not been clarified, psychological suffering due to sensory symptoms may increase internalizing problems. Sensory symptoms can cause various problems in daily life. For example, Thye et al. (2018) reviewed the impact of sensory symptoms on social functions and claimed that they could cascade into social deficits across development. Furthermore, some sensory symptoms have been associated with academic underachievement in children with ASD in regular education classes (Ashburner et al., 2008). In addition, adolescents with ASD attending mainstream schools reported that sensory symptoms affected their learning and caused various negative feelings such as anxiety and emotional and physical discomfort (Howe and Stagg, 2016). Parents and teachers also perceived that autistic children’s distress while at school was due to sensory symptoms (Jones et al., 2020). Thus, individuals with ASD seem to suffer from problems caused by sensory symptoms.

Chronic stress is a prolonged imbalance between situational requirements and the individual’s coping resources (Colombo et al., 2020) and can lead to anxiety and depression (McGonagle and Kessler, 1990; Khan and Khan, 2017). Prolonged suffering due to sensory symptoms or other autistic traits can be a chronic stressor and cause internalizing problems such as anxiety and depression. Indeed, some studies reported that subjective suffering due to autistic traits is associated with depression and anxiety (Shinoda et al., 2017; Harada, 2019). Similarly, a previous study of university students suggested that those who scored high for sensory symptoms demonstrated subjective suffering due to sensory symptoms in universities as a predictor of internalizing problems (Tsuji et al., 2022). Thus, internalizing problems may occur when individuals suffer from sensory symptoms.

Support to decrease suffering due to sensory symptoms, rather than the symptoms themselves, can effectively reduce internalizing problems. For example, Sensory Profile (SP; Dunn, 1999), which is used widely to measure sensory symptoms, includes the following items: “twirling/spinning self throughout the day (for example, likes dizzy feeling)” and “responds negatively to unexpected or loud noises (for example, cries or hides at noise from vacuum cleaner, dog barking, hair dryer).” If children enjoy spinning, it may not cause much suffering. In contrast, students with ASD reported that auditory symptoms most affected their learning and caused negative feelings (Howe and Stagg, 2016). Therefore, spinning oneself as a preferred behavior will not increase internalizing problems, but they may be increased by loud noises, promoting negative responses such as avoidance. In such cases, we should prioritize noise control, even if spinning is also one of the sensory symptoms. In this way, increased understanding of the suffering of children with ASD may motivate those around them to find ways to provide support. Therefore, it is necessary to focus on the frequency of responses to sensory experiences and the suffering arising from atypical reactions to these.

Additionally, even if children with sensory symptoms do not suffer, people in their proximity usually do. For example, if a child cannot sit still during class, the class must often stop for the child, and teachers and classmates may suffer. Thus, without appropriate support, children’s relationships with those around them may deteriorate, causing them to experience internalizing problems. Therefore, it is necessary to investigate children’s suffering due to sensory symptoms and the degree to which people around them suffer. This information will clarify when and where support is needed.

In the present study, we developed and administered a scale to assess the degree of suffering due to sensory symptoms in daily experiences, especially school life, for elementary and junior high school students. Furthermore, we tested whether internalizing problems were positively associated with sensory symptoms and suffering due to sensory symptoms in children and the people in their school environment. Thus, we created a hypothetical model in which sensory symptoms were indirectly related to internalizing problems *via* suffering due to sensory symptoms. This study provides an essential contribution by elucidating the suffering caused by sensory symptoms to provide more appropriate support in schools.

## Materials and methods

### Participants

The participants included 113 students enrolled in elementary or junior high school in Japan (53 boys, 57 girls, 3 gender unknown). The mean age was 10.59 years (*SD* = 2.43, range = 6–15). Of the participating children, 25 were diagnosed with ASD (22 boys, 2 girls, 1 gender unknown; mean age of 10.64 years, *SD* = 2.60, range = 6–15). A psychiatrist (the last author) recruited them at a psychiatric clinic or center for clinical psychology. Of the participating children without ASD (*n* = 88), 77 included in the TD group were recruited in cram schools or music classes (26 boys, 49 girls, 2 gender unknown; mean age of 10.53 years, *SD* = 2.42, range = 6–15). Those who were diagnosed with developmental disability other than ASD (*n* = 6), or were not diagnosed but did not attend regular class (*n* = 5), were not included in the ASD or TD groups. Therefore, all children in the TD group were not diagnosed as developmental disability and attended regular classes.

Parents of the participating children (105 mothers, 6 fathers, and 2 parents) completed the battery of measures. All parents provided written informed consent prior to participating in the study. This study was approved by the ethics committee of Kitasato University School of Allied Health Sciences and conducted in accordance with the Declaration of Helsinki.

### Measures

#### Demographics

Demographic information including age, gender, class type of school (e.g., regular class, special needs class, and special needs school), the presence/absence of the diagnosis of developmental disorders, and the diagnostic names were obtained through parent reports.

#### Autistic traits

To measure autistic traits, we used the Japanese version of the Child Autism-Spectrum Quotient (AQ-Child), which is a 50-item parent-report questionnaire for children aged 6–15 years (Auyeung et al., 2008; Wakabayashi, 2016). The standardization study showed that 85.2% of the ASD group scored a cutoff of 25 or more, although only 3.8% of the control group scored in this range (Wakabayashi et al., 2007). In this study, Cronbach’s alpha was 0.89.

#### Sensory symptoms

The Japanese version of the Sensory Profile (SP) for individuals aged 3–82 years was used to measure sensory symptoms (Hagiwara et al., 2015). This is a 125-item parent-report questionnaire on functional behaviors associated with atypical responses to sensory stimuli. The original version of the SP was developed based on Dunn’s model (Dunn, 1997, 1999). The SP items are grouped into three categories: Sensory Processing, Modulation, and Behavioral and Emotional Responses. Sensory Processing is further categorized into six domains: Auditory, Visual, Vestibular, Touch, Multisensory, and Oral Sensory Processing. Items from each category were incorporated into four distinct quadrants to characterize experience and behavior: Low Registration, Sensation Seeking, Sensory Sensitivity, and Sensation Avoiding. Cronbach’s alpha in the current study was between 0.92 and 0.95 for each category, between 0.82 and 0.92 for each quadrant, and 0.97 for all 125 items.

#### Suffering due to sensory symptoms in school

We developed eight items to measure the children’s and surrounding people’s psychological suffering due to sensory symptoms in school life. These items were developed based on the Sensory Processing category of the SP, and each of the two items can be classified into four quadrants. For example, the item “Sometimes appears to not hear what teacher says in class” was developed from “Appears to not hear what you say” included in the auditory domain and Low Registration. For each item, parents evaluated the extent to which their children and those around them (i.e., teacher and classmates) suffered from the children’s sensory symptoms in school life, respectively. In other words, parents evaluated how much their children suffered in each situation. They answered the same questions again about the discomfort of those around them. The state of suffering meant that children or those around them suffer because they cannot cope well with the children’s sensory symptoms. Therefore, this scale was intended to assess separately the degree of psychological suffering experienced by the children and those around them. The SP measured the frequency of sensory experiences.

Responses were scored on a four-point Likert scale (0 = *do not suffer*, 1 = *suffer a little*, 2 = *suffer*, 3 = *suffer much*) for the children and those around them. Higher scores indicate greater suffering due to sensory symptoms in schools. All items are presented in Table 1 and the Supplementary material.

**Table 1:** PCA of suffering due to sensory symptoms.

Items	Loading	
	Children’s suffering (*n* = 112)	Surrounding people’s suffering (*n* = 106)
*Suffering due to Low Registration*		
1. Sometimes appears not to hear what teacher says in class.	0.74	0.77
2. Is oblivious within an active environment and it makes it difficult to follow the class.	0.73	0.68
*Suffering due to Sensory Seeking*		
3. Fidgets and moves around during class.	0.79	0.81
4. Touches people and objects and other children do not like it.	0.59	0.80
*Suffering due to Sensory Sensitivity*		
5. Cannot concentrate on class because is distracted or has trouble functioning if there is noise.	0.71	0.78
6. Avoids certain tastes or food smells, so cannot eat many items in school lunch or always eats the same thing for lunch.	0.43	0.36
*Suffering due to Sensation Avoiding*		
7. Cannot attend school events or activities because dislike unexpected or loud noises.	0.67	0.63
8. Sometimes dislikes or gets angry because of being touched by other people.	0.62	0.78
Explained variance	44.89%	51.01%

#### Internalizing problems

The Japanese version of the Strength and Difficulties Questionnaire (SDQ) for those aged 4–17 is a 25-item parent-report questionnaire (Goodman, 1997; Youthinmind, 2020). Each of the five subscales included five items. We used two subscales: emotional symptoms and peer problems, to measure internalizing problems. These subscales measure internalizing problems that appear as intropunitive emotional states such as anxiety and depression that reflect internal distress and behavioral states that involve difficulties in relationships with other children. There is theoretical and preliminary empirical support for combining emotional symptoms and peer problems into an internalizing subscale (Goodman et al., 2010), and this is widely used. In this study, the total score of 10 items was used to indicate the intensity of internalizing problems. Cronbach’s alpha in the current study was 0.80, 0.74, and 0.81 for emotional symptoms, peer problems, and the total score, respectively.

### Statistical analysis

Using principal component analysis (PCA), we investigated the factor structure of the suffering due to sensory symptoms in schools among children and people around them. We further investigated the distribution of autistic traits and sensory symptoms in children with TD (*n* = 77) and children with ASD (*n* = 25) to confirm the analysis validity.

As the TD group had more girls, we used t-tests to check whether there were gender differences in the group’s variables.

Children with TD and ASD who attended regular classes without missing values (*n* = 69) were included to investigate children in similar school environments. The correlation coefficients were calculated between autistic traits, sensory symptoms (i.e., score of each domain in the Sensory Processing category in the SP), suffering due to sensory symptoms for children and surrounding people, and internalizing problems (i.e., two subscales of SDQ).

Moreover, structural equation modeling (SEM) was used to verify the hypothetical model that sensory symptoms indirectly affect internalizing problems *via* suffering due to sensory symptoms. We used the score in the Sensory Processing category, excluding the visual domain as the index of sensory symptoms. We also tested the mediating effects of suffering due to sensory symptoms using 5,000 bootstrap samples (Mallinckrodt et al., 2006).

Data analysis was performed using SPSS 25.0 and Amos 23.0 (IBM Corp., Armonk, New York). The dataset for this analysis is available in the Supplementary material.

## Results

### Principal component analysis of suffering due to sensory symptoms

We examined the factor structure and internal consistency of the eight items regarding suffering due to sensory symptoms in schools among children and the people around them. This analysis included children with TD and ASD without missing values. Regarding children’s suffering, PCA revealed that the first factor explained 44.89% of the total variance, with an eigenvalue of 3.59, and all remaining factors had eigenvalues 1.03 or less. Regarding surrounding people’s suffering, the first factor explained 51.01% of the total variance, with an eigenvalue of 4.08, and all remaining factors had eigenvalues 1.05 or less. All eight items had factor loadings of.36 or higher for the first factor in the children’s and surrounding people’s suffering (Table 1). Therefore, the single-factor model was confirmed as appropriate for both children and surrounding people. Cronbach’s alpha was 0.79 for suffering for children and 0.85 for surrounding people. Therefore, the total score for the items was used in the analysis to represent suffering due to sensory symptoms among the children and people around them.

### Gender differences in the typical development group

As TD group had more girls, we used *t*-tests to investigate the gender differences in TD group. There were no gender difference for the total AQ score (*t*_(64)_ = 0.87, *p* = 0.40, *d* = 0.22), total SP score (*t*_(66)_ = 0.84, *p* = 0.41, *d* = 0.21), and the total score of SDQ items (*t*_(70)_ = 0.74, *p* = 0.46, *d* = 0.18). Regarding the suffering due to sensory symptoms, although there were no gender difference for the children’s suffering (*t*_(72)_ = 0.23, *p* = 0.82, *d* = 0.06), the surround people’s suffering was higher for boys than for girls (*t*_(31.72)_ = 2.20, *p* = 0.03, *d* = 0.59). Therefore, there was no gender difference for variables except for surround people’s suffering.

### Distribution of autistic traits and sensory symptoms

**Figure 1:**
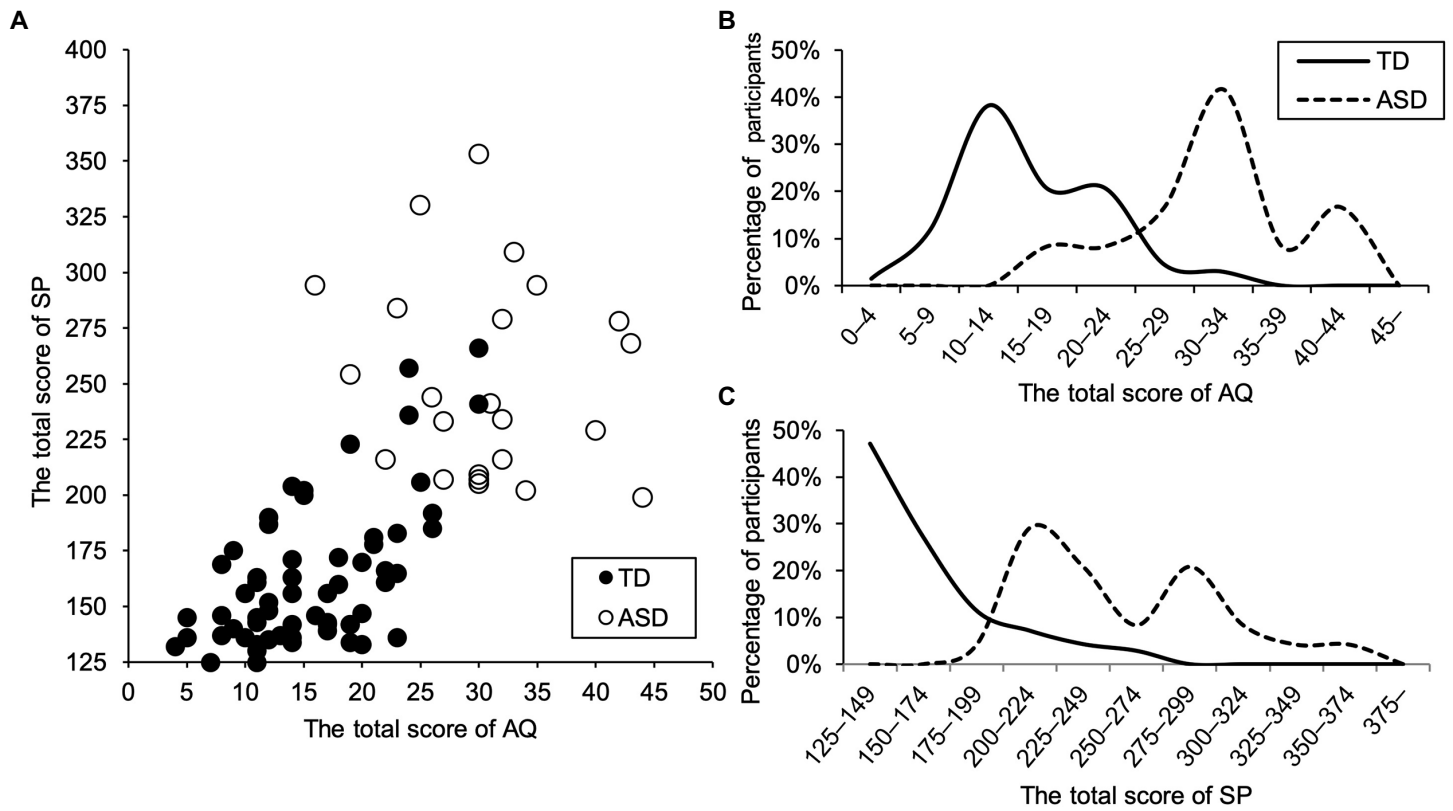
Frequency distribution **(B and C)** and scatter plot **(A)** of autistic traits and sensory symptoms in TD group (*n* = 77) and ASD group (*n* = 25).

Results showed that 83.3% (*n* = 20) of the ASD group scored a cutoff of 25 or more on the total AQ-Child score, and only 7.4% (*n* = 5) of the TD group scored in this range. However, the scores of both groups exhibited heavy-tailed overlapping distributions (Figure 1). Therefore, although the scores of autistic traits were higher in the ASD group than in the TD group, the traits were distributed as a continuum throughout both groups.

In the ASD group, 20.0, 29.2, 36.0, and 16.0% of children scored within the average range on Low Registration, Sensory Seeking, Sensory Sensitivity, and Sensation Avoiding in SP, respectively. In contrast, 79.2, 91.8, 87.5, and 84.7% of children with TD scored within the average range on Low Registration, Sensory Seeking, Sensory Sensitivity, and Sensation Avoiding, respectively. Thus, most children with ASD had higher SP scores. They showed high sensory symptoms, while most children with TD did not. However, the total SP scores in the two groups also exhibited heavy-tailed overlapping distributions (Figure 1).

### Relationships between each variable

The school environment for children in special needs classes and special needs schools is quite different from regular classrooms. This difference may affect the suffering or internalizing problems. Hence, the following analysis included 69 children with TD and ASD who attended regular classes without missing values to investigate the relationships between each variable among children in similar school environments.

There were significant positive correlations between suffering due to sensory symptoms for the children and surrounding people, each domain of Sensory Processing, and subscales of internalizing problems (see Supplementary material).

**Figure 2:**
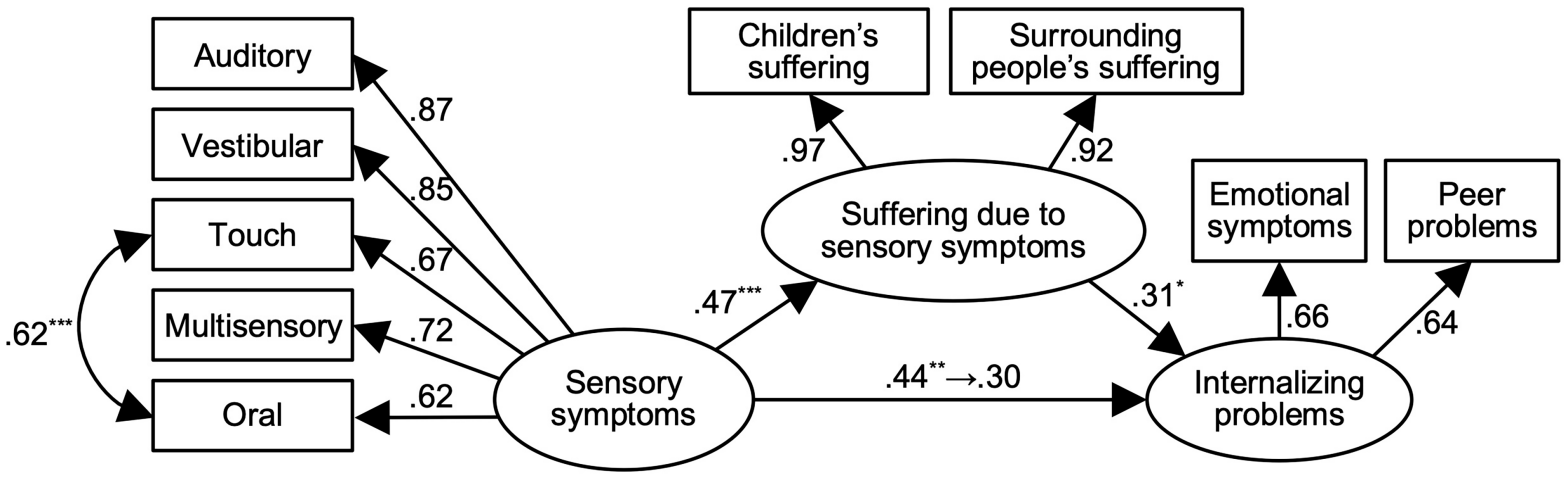
SEM was performed for children attending regular classes (*n* = 69), χ^2^(2) = 24.35, *p* = 0.71, CFI = 1.00, RMSEA = 0.00. Autistic traits were controlled. The results of significance test only for path coefficients between sensory symptoms, suffering due to sensory symptoms, and internalizing problems and correlation coefficient between Touch and Oral were described. ^*^p < 0.05, ^**^p < 0.01, ^***^p < 0.001.

SEM was then used to evaluate the relationship between variables. We built the hypothetical model that sensory symptoms indirectly affect internalizing problems *via* the suffering they cause. SEM is suited for hypotheses involving mediators because this analytic technique can test direct and indirect effects (Coffey and Hartman, 2008). Moreover, SEM tests a model that represents the relationships between latent variables and observed variables (Mayfield and Mayfield, 2008). As our model involved a mediator and latent variables, we verified our model using this technique. The index of suffering due to sensory symptoms items was developed based on the Sensory Processing category excluding the visual domain. Therefore, we excluded it from the SEM. The scores for autistic traits were used as control variables. The model provided a good fit to the data (Figure 2; *χ*^2^_(2)_ = 24.35, *p* = 0.71, CFI = 1.00, RMSEA = 0.00).

Both the path coefficient from sensory symptoms to suffering due to sensory symptoms (*β* = 0.47, *p* < 0.001) and that from suffering due to sensory symptoms to internalizing problems (*β* = 0.31, *p* < 0.05) were significant. As hypothesized, these results suggest a partial mediation of suffering due to sensory symptoms between sensory symptoms and internalizing problems. Therefore, we investigated the mediating effects of suffering due to sensory symptoms. The direct effect of sensory symptoms on internalizing problems decreased from.44 (*p* < 0.01) to 0.30 (*n.s.*) when the suffering due to sensory symptoms were added as a mediator. Furthermore, the 95% confidence interval of the indirect effect based on 5,000 bootstrap samples was −0.03 to 0.28, suggesting that the indirect effect was marginally significant.

## Discussion

We conducted a cross-sectional study of students with and without ASD to investigate whether the relationship between sensory symptoms and internalizing problems was mediated by the suffering due to sensory symptoms.

### Distribution of autistic traits and sensory symptoms

Although the scores for autistic traits and sensory symptoms were higher in the ASD group than in the TD group, these traits were distributed on a continuum throughout both groups. The TD group included individuals with high autistic traits and sensory symptoms. The results indicate that autistic traits and sensory symptoms were distributed on a continuum throughout the general population. This finding is consistent with previous studies (Robertson and Simmons, 2013; Kamio et al., 2013a). Regardless of ASD diagnosis, individuals with high autistic traits have difficulty adapting to society. Without adequate support, their quality of life often decreases (Kamio et al., 2013b). These results suggest the importance of support for individuals who were not diagnosed with ASD but have high autistic traits. Therefore, excluding children without an ASD diagnosis is unreasonable. An investigation of the relationship between sensory symptoms and internalizing problems among children with and without ASD is valid.

### Relationships between each variable in children attending regular classes

SEM revealed that psychological suffering due to sensory symptoms partially mediated the relationship between sensory processing and internalizing problems in children attending regular classes. These results are consistent with a previous study suggesting that the suffering due to sensory symptoms mediate the relationship between sensory processing and internalizing problems among general university students (Tsuji et al., 2022). This mediation role was apparent in children attending regular classes. Although Tsuji et al. (2022) showed only the mediation role of university students’ suffering, the present study demonstrated the mediating role of suffering for the children and surrounding people in elementary and junior high schools. Our quantitative results thus support qualitative results indicating that there are problems due to sensory symptoms in mainstream schools (Howe and Stagg, 2016).

Sensory symptoms cause various problems in school (Ashburner et al., 2008; Howe and Stagg, 2016). However, according to Kasahara (2010), issues related to sensory symptoms are often misunderstood or underestimated. Therefore, teachers may lack awareness of the children’s sensory problems. Children suffer from these problems without enough support. Moreover, difficulties related to sensory symptoms, such as children’s behaviors that interfere with classwork, can also increase the suffering for teachers or other children. In addition, the high degree of suffering of people around them leads to deterioration in their relationship with teachers and other children.

The children’s suffering leads to internalizing problems. Since chronic stress can induce anxiety and depression (McGonagle and Kessler, 1990; Khan and Khan, 2017), children’s suffering due to sensory symptoms and deterioration of social relationships due to surrounding people’s suffering may act as chronic stressors, causing internalizing problems.

### Support for children

Suffering from sensory symptoms mediated the relationship between sensory processing and internalizing problems for children attending regular classes. Therefore, the internalizing problems of these children, especially those in regular classes, may be reduced by providing support that decreases suffering even if sensory symptoms are not entirely eliminated.

In addition, this study included a few children who were not diagnosed with ASD but had high autistic traits and sensory symptoms. Therefore, support focused on reducing psychological suffering may be effective for children with and without ASD. Although SP is widely used to measure sensory symptoms, caregivers or teachers probably should also use a scale that evaluates the suffering due to sensory symptoms to understand children’s suffering and find what supports are needed for children with sensory symptoms. As inclusive education promotes mixing children with and without ASD learning in the same classroom, these suggestions related to regular classes are essential for caregivers or teachers to provide adequate support.

### Limitations

We conducted an exploratory study on the suffering due to sensory symptoms, including differences in school environments. However, since the sample was small, the investigations regarding age and sex were insufficient. Further large sample studies are needed to yield more robust findings regarding support for suffering due to sensory symptoms.

In addition, this study indicated that the relationship between sensory symptoms and internalizing problems was mediated by the suffering due to sensory symptoms. However, we could not investigate the relationship between sensory symptoms and internalizing problems in children attending special needs classes and special needs schools.

A scale to evaluate the suffering due to sensory symptoms for children has not been developed previously. Hence, it is meaningful that we developed a new scale to evaluate the suffering due to sensory symptoms in children and the people around them. However, their parents evaluated their children’s suffering. A self-report scale is necessary to investigate this aspect more accurately. Similarly, it will be useful for a person familiar with the child’s behaviors, such as their schoolteacher, to evaluate the suffering among surrounding people. It is also necessary to collect more items through interviews with the children and those around them and investigate the suffering in daily life inside and outside school. Furthermore, although the developed scale of suffering was related to SP and had some convergent validity, an examination of divergent validity and test–retest reliability is needed.

## Conclusion

This study showed that suffering due to sensory symptoms in school life mediated the relationship between sensory symptoms and internalizing problems in children with and without ASD. Since suffering due to sensory symptoms for children and those around them can interrupt their education, it is necessary to develop and provide adequate support for students. Further research on suffering due to sensory symptoms in various populations and investigations of effective support will enable us to provide better support for people with sensory symptoms.

## Data availability statement

The original contributions presented in the study are included in the article/Supplementary material, further inquiries can be directed to the corresponding author.

## Ethics statement

The studies involving human participants were reviewed and approved by Kitasato University School of Allied Health Sciences. Written informed consent to participate in this study was provided by the participants’ legal guardian/next of kin.

## Author contributions

YT, MS, and AO conceived the study. YT and AO collected the data. YT and SI analyzed the data. YT drafted the manuscript. All authors revised the manuscript and approved the final version of the manuscript.

## Funding

This study was supported by a funding from Kitasato University and Grant-in-Aid for Early-Career Scientists (20K20144) from the Japan Society for the Promotion of Science. The funders had no role in study design, data collection and analysis, interpretation of results, manuscript preparation, or decision to submit for publication.

## Conflict of interest

The authors declare that the research was conducted in the absence of any commercial or financial relationships that could be construed as a potential conflict of interest.

## Publisher’s note

All claims expressed in this article are solely those of the authors and do not necessarily represent those of their affiliated organizations, or those of the publisher, the editors and the reviewers. Any product that may be evaluated in this article, or claim that may be made by its manufacturer, is not guaranteed or endorsed by the publisher.
