# Supplementary material for: Internalizing problems and suffering due to sensory symptoms in children and adolescents with and without autism spectrum disorder
Source: Front Psychol. 2022 Aug 5;13:872185. doi: 10.3389/fpsyg.2022.872185 (PMC9390977; doi:10.3389/fpsyg.2022.872185)
Supplement: Supplementary file 1 [file Table_1.PDF]

Questionnaire of suffering due to sensory symptoms

あなたのお子さんが学校生活を送る上で、それぞれの項目の内容について、お子さん自身がどの程度困っているかと、お子さんの周囲の人 (学校の先生や他の生徒など) がどの程度困っているかをそれぞれ判断し、最もあてはまる数字に 1 つずつ〇をつけてください。

0：困っていない
1：少し困っている
2：困っている
3：とても困っている

	お子さん自身の困り感	周囲の人 (学校の先生や他の生徒など) の困り感
1. 授業中、先生が話しかけても聞いていないことがある	0 － 1 － 2 － 3	0 － 1 － 2 － 3
2. 活動的な環境の中でぼんやりしていて、授業についていかれないことがある	0 － 1 － 2 － 3	0 － 1 － 2 － 3
3. 授業中でも落ち着きがなかったり、動きまわってしまう	0 － 1 － 2 － 3	0 － 1 － 2 － 3
4. 他の子が嫌がるほど人や物にさわりたがる	0 － 1 － 2 － 3	0 － 1 － 2 － 3
5. まわりの音のせいで気が散ったりうまく活動できなくなり、授業に集中できないことがある	0 － 1 － 2 － 3	0 － 1 － 2 － 3
6. 味や食感、においが嫌いなものが多いので、給食で食べられないものが多かったり、お弁当の中身がいつも同じものばかりになる	0 － 1 － 2 － 3	0 － 1 － 2 － 3
7. 突然の、または大きな音 (例：運動会でのピストルの音など) が苦手なせいで、学校での活動に支障が出たり、参加できないことがある	0 － 1 － 2 － 3	0 － 1 － 2 － 3
8. 他の子や先生にさわられたことで、嫌がったり怒ったりすることがある	0 － 1 － 2 － 3	0 － 1 － 2 － 3
