# Supplementary material for: Internalizing problems and suffering due to sensory symptoms in children and adolescents with and without autism spectrum disorder
Source: Front Psychol. 2022 Aug 5;13:872185. doi: 10.3389/fpsyg.2022.872185 (PMC9390977; doi:10.3389/fpsyg.2022.872185)
Supplement: Supplementary file 2 [file Table_2.PDF]

Correlations between autistic traits, sensory symptoms, suffering due to sensory symptoms, and internalizing problems in children with TD and ASD attending regular classes (n = 69).

	Autistic traits	Suffering due to sensory symptoms		Internalizing problems	
		Children's suffering	Surrounding people's suffering	Emotional symptoms	Peer problems
Sensory symptoms					
Auditory	$.51^{***}$	$.57^{***}$	$.56^{***}$	$.52^{***}$	$.48^{***}$
Visual	$.34^{**}$	$.30^{*}$	$.29^{*}$	$.41^{***}$	$.31^{**}$
Vestibular	$.57^{***}$	$.54^{***}$	$.56^{***}$	$.53^{***}$	$.46^{***}$
Touch	$.41^{***}$	$.49^{***}$	$.48^{***}$	$.30^{*}$	$.43^{***}$
Multisensory	$.55^{***}$	$.42^{***}$	$.41^{***}$	$.46^{***}$	$.46^{***}$
Oral Sensory	$.44^{***}$	$.47^{***}$	$.42^{***}$	$.28^{*}$	$.42^{***}$
Suffering due to sensory symptoms					
Children's suffering	$.62^{***}$				
Surrounding people's suffering	$.53^{***}$	$.89^{***}$			
Internalizing problems					
Emotional symptoms	$.68^{***}$	$.53^{***}$	$.48^{***}$		
Peer problems	$.61^{***}$	$.60^{***}$	$.58^{***}$	$.42^{***}$	

Note. $^{*}p < .05$, $^{**}p < .01$, $^{***}p < .001$
